# Collagen-Carboxymethylcellulose Biocomposite Wound-Dressings with Antimicrobial Activity

**DOI:** 10.3390/ma14051153

**Published:** 2021-03-01

**Authors:** Ionela Andreea Neacsu, Sorina-Alexandra Leau, Stefania Marin, Alina Maria Holban, Bogdan-Stefan Vasile, Adrian-Ionut Nicoara, Vladimir Lucian Ene, Coralia Bleotu, Mădălina Georgiana Albu Kaya, Anton Ficai

**Affiliations:** 1 Faculty of Applied Chemistry and Materials Science, University Politehnica of Bucharest, 060042 Bucharest, Romania; neacsu.a.ionela@gmail.com (I.A.N.); sorina.leau@yahoo.com (S.-A.L.); bogdan.vasile@upb.ro (B.-S.V.); adi.nicoara18@gmail.com (A.-I.N.); anton.ficai@upb.ro (A.F.); 2 National Research Center for Micro and Nanomaterials, University Politehnica of Bucharest, 060042 Bucharest, Romania; 3 Electrochemistry and Corrosion Department, “Ilie Murgulescu” Institute of Physical Chemistry, Romanian Academy, 060021 Bucharest, Romania; 4 INCDTP-Division: Leather and Footwear Research Institute, 93 Ion Minulescu Str., 011061 Bucharest, Romania; marinstefania.92@gmail.com (S.M.); albu_mada@yahoo.com (M.G.A.K.); 5 Microbiology and Immunology Department, Faculty of Biology, Research Institute of the University of Bucharest, University of Bucharest, 060101 Bucharest, Romania; alina_m_h@yahoo.com; 6 Stefan S. Nicolau’ Institute of Virology, Romanian Academy, 011061 Bucharest, Romania; cbleotu@yahoo.com

**Keywords:** wound-dressings, collagen, carboxymethylcellulose, silver nanoparticles

## Abstract

Microbial infections associated with skin diseases are frequently investigated since they impact on the progress of pathology and healing. The present work proposes the development of freeze-dried, glutaraldehyde cross-linked, and non-cross-linked biocomposite dressings with a porous structure, which may assist the reepithelization process through the presence of collagen and carboxymethylcellulose, along with a therapeutic antimicrobial effect, due to silver nanoparticles (AgNPs) addition. Phisyco-chemical characterization revealed the porous morphology of the obtained freeze-dried composites, the presence of high crystalline silver nanoparticles with truncated triangular and polyhedral morphologies, as well as the characteristic absorption bands of collagen, silver, and carboxymethylcellulose. In vitro tests also assessed the stability, functionality, and the degradability rate of the obtained wound-dressings. Antimicrobial assay performed on Gram-negative (*Escherichia coli*), Gram-positive (*Staphyloccocus aureus*) bacteria, and yeast (*Candida albicans*) models demonstrated that composite wound dressings based on collagen, carboxymethylcellulose, and AgNPs are suitable for skin lesions because they prevent the risk of infection and have prospective wound healing capacity. Moreover, the cell toxicity studies proved that the obtained materials can be used in long time treatments, with no cytotoxic effects.

## 1. Introduction

The skin is the largest organ of the human body, and it accounts for about 15% of the body’s weight, averages 1.8 m^2^ in surface, and has a thickness of 1.5–4 mm [1,2]. The main function of skin is to protect the interior of the body from the external environment, and it performs this role in many ways: It acts as a semi-permeable membrane for both hydrophilic and hydrophobic substances, it is the first immunological defense line against microbes and it is involved in the metabolism of vitamin D [3]. When the integrity of the skin is compromised, most often from thermal (burns) or mechanical causes, the wound becomes a favorable place for microbial proliferation and colonization. The affected area can be colonized by various Gram-positive (i.e., *Staphylococcus aureus*, *Streptococcus* sp., *Micrococcus* sp.) and Gram-negative (i.e., *Escherichia coli*, *Pseudomonas aeruginosa*) bacteria, and sometimes yeasts (i.e., *Candida albicans*) [4].

Classical treatment of infections is based on systemic antibiotic administration for large period of time, usually leading to complications because of low specificity (the drug is dispersed throughout the entire organism and impacts on resident microbiota), low efficiency (only a small amount of the dispersed drug reaches the infected area, especially in localized infections), and to the increased selection of bacterial resistance. The best way of avoiding such situation is to develop effective preventive methods based on antibacterial systems able to deliver antibiotics, antimicrobial peptides, natural extracts, essential oils, etc., in situ, at the infection site [5,6]. This approach of locally delivering the active substances presents a series of advantages, including high efficacy at low dose, the possibility of simultaneously delivering one or more drugs that can work synergistically, a stable drug concentration in the therapeutic range, and a lower occurrence of side effects [7].

Hydrogels represent such a delivery system, easy to obtain from almost every hydrophilic polymer, with varying porosity, density, absorbability, and therefore release rate. Hydrogels can be used as coating for various medical devices (catheters, lenses, stents, bone implants), as well as for wound healing and drug release. Their usage as antimicrobial wound-dressings is due to the inherent antimicrobial properties some polymers possess [8], or to the addition small amounts of antibacterial agents [9,10]. Among the hydrophilic macromolecules used as hydrogels, collagen, and sodium carboxymethylcellulose are both recently studied due to their unique properties that make them suitable as a polymer matrix for wound-dressings. Namely, collagen (Coll) is a biodegradable and biocompatible protein produced by fibroblasts, mostly found in the connective tissue and exhibits similarities to human skin in terms of composition and structure. It provides structural support to the organism and regulates cellular functions such as cellular formation, proliferation, differentiation, and cellular migration. Collagen absorbs exudate, but also has an important function in the natural healing process of wounds by inducing coagulation and scar formation. If collagen is combined with dermal cells, growth factors, and cytokines in the patient’s body, the reepithelization process is accelerated [11,12,13]. In addition, sodium carboxymethylcellulose (CMC) also exhibits high affinity for water, has a low price and excellent skin compatibility (it’s physiologically harmless), being already used in treatment devices, especially for burns, being also able to assure the desired mechanical strength. In the lesion area, it maintains an optimal humid medium for extracellular matrix formation and reepithelization to take place. The moist medium has certain advantages, namely that it prevents the dehydration of the tissue and its necrotizing, relieves the sensation of pain, promotes angiogenesis, and removes the necrotic tissue if the lesion is in an advanced state of decay [14,15].

When selecting a suitable antimicrobial agent to add into the hydrogel, some aspects must be taken into consideration, usually referring to the pathogens causing the infection, the site of the infection, the pharmacokinetic (tissues penetration, elimination mode, etc.), pharmaceutical (preparation, use, etc.) properties, and safety profile of the agent [16,17]. Even though antibiotics are increasing life expectancy in developing countries, a series of side effects emerge from the excessive consumption, over/underuse, and misuse of antibiotics, a major challenge being bacterial resistance [18]. In order to overcome the antibiotic resistance, an immediate solution seems to be the delivery of a lower dose of the already existing bactericides directly to the infected area, or, even better, the delivery of nanoparticles with antimicrobial activity [7,19]. Metallic and oxide nanoparticles (Ag, ZnO, MgO, CuO, etc.) loaded hydrogels are widely investigated, being biocompatible, bioresorbable, and easy to produce and deliver in different forms [20,21,22]. Nanostructured silver particles exhibit unique physical and chemical properties that are even more suitable for biomedical applications than those of silver in any other formula. These properties make silver nanoparticles (AgNP) efficient materials in controlling the progression of various severe infections (e.g., hospital acquired infections, wound infections) [23,24].

Despite the large number of scientific reports based on wound dressing materials, including collagen, sodium carboxymethylcellulose or silver nanoparticles, there are limited studies on the usage of these particular 3 components altogether to obtain glutaraldehyde cross-linked and non-cross-linked porous composites with enhanced antimicrobial activity [25].

## 2. Materials and Methods

### 2.1. Materials

The materials used for the colloidal silver and final biocomposites production were: Silver nitrate (AgNO_3_, *p* ≥ 99.0%), citric acid (C_6_H_5_O_8_, *p* ≥ 99.5%), polyvinylpyrrolidone (PVP, K-30), sodium borohydride (NaBH_4_, *p* = 99%), sodium hydroxide (NaOH, *p* ≥ 98%), sodium carboxymethylcellulose (C_8_H_11_O_7_Na, average M_W_ ~250.000 Da), collagenase type I, glutaraldehyde (C_5_H_8_O_2_, 25% solution in water), and hydrogen peroxide solution (30% (*w/v*) in H_2_O). They were procured from Sigma-Aldrich, Darmstadt, Germany and used without further purification. Type I collagen gel was produced at the Research-Development Textile Leather National Institute Division Leather and Footwear Research Institute—Collagen Department, Bucharest, Romania, and used after dilution.

### 2.2. AgNPs Synthesis

In order to obtain a 100 ppm colloidal silver nanoparticle solution, a chemical reduction method was used, derived from the aqueous solution phase route of Zielinska et al. [26]. This method involves the reduction of Ag^+^ to Ag^0^, starting from AgNO_3_ as silver precursor and a reducing mixture based on the synergistic effect of NaBH_4_ and citric acid (biocompatible antioxidant, similar with ascorbic acid, but with a reducing effect known to be lower than that of NaBH_4_ [27]). Citrate ions also play the roles of stabilizing and complexing agent, and it is reasonable to assume that silver citrate complexes would act synergistically as antimicrobial agents and antioxidants [28]. Briefly, 100 mL of AgNO_3_ 10^−4^ M were mixed with 6 mL of C_6_H_5_O_8_ 0.3 M and 600 µL of NaBH_4_ 0.1 M. The obtained mixture was vigorously stirred at room temperature on a magnetic stirrer and after 12 min 6 mL of PVP K-30 7 × 10^−3^ M were added. The reason for introducing PVP was to prevent the AgNPs from growing and agglomerating, and to better control the final morphology [29]. In the final step, 140 mL of H_2_O_2_ 30% were added, and the mixture was stirred for about 10 min until a deep blue color was obtained, an indication of the nanoparticles reduced dimension [30].

### 2.3. Biocomposite Materials Synthesis

Collagen-carboxymethylcellulose composite materials were obtained starting from a type I fibrillary collagen gel with a concentration of 2.26% (*w/w*), extracted from calf hide [31]. The received gel was afterwards adjusted in terms of concentration and pH (to 1% w/w and physiological medium human body pH of 7.4) using NaOH 1M. CMC powder was then added to the mixture, in the same amount as the dry content from the collagen gel (1%) and stirrer until homogenization. The obtained gel was further divided into 6 samples and mixed with various amounts of 100 ppm colloidal silver nanoparticle solution (10–20 mL) and/or glutaraldehyde aqueous solution 0.025% and maintained over night at 4 °C, resulting in the samples from Table 1.

After crosslinking, the samples denoted with “G” were saturated in distilled water and preserved for several hours, in order to remove the unreacted glutaraldehyde. This process was repeated until a negative result was obtained on the Fehling test, then all samples were freeze-dried using a Delta 2-24 DSC lyophilizer (Martin Christ, Osterode am Harz, Germany) with a 3-step 48 h lyophilization program (cooling until −40 °C/atmospheric pressure, followed by a pressure decrease down to 0.01 mbar for 12 h, then heating under vacuum for 24 h up to 35 °C). Each porous material consisted of equal mass of dry polymers (0.5 g Coll, 0.5 g CMC) and 0 mg, 0.1 mg, or 0.2 mg of AgNPs, respectively, depending on the added volume of the colloidal solution.

### 2.4. Structural and Morphological Analyses

The biocomposites specific morphology due to the freeze-drying process, the distribution and pore size, as well as the dispersion of silver nanoparticles on the polymers fibers were investigated via Scanning Electron Microscopy (SEM), using an Inspect F50 high resolution microscope coupled with an energy dispersive spectrometer (EDS) (Thermo Fisher—former FEI, Eindhoven, Netherland). The acquisition of micrographs was made with an energy value of 30 KeV at different magnifications. Transmission Electron Microscopy (TEM) was used to obtain important information related to the morphology and crystallinity of the silver nanoparticles. TEM images for the colloidal silver were obtained with a high resolution transmission electron microscope, Tecnai G2 F30 S-TWIN (Thermo Fisher—former FEI, Eindhoven, Nederland), equipped with selected area electron diffraction (SAED) module. This microscope operates in transmission mode at a voltage of 300 kV, the punctual resolution and the guaranteed line having the values of 2 Å and 1 Å, respectively.

Fourier Transform Infrared Spectroscopy (FT-IR) involved the analysis of biocomposite wound-dressings via Nicolet iS50R spectrometer (Thermo Fisher, Waltham, MA, USA). The measurements were performed at room temperature, with 32 sample scans between 4000 cm^−1^ and 400 cm^−1^, at a resolution of 4 cm^−1^. Spectral data recording was possible by connecting the spectrometer to a data collection and processing unit, through the Omnic work program. The purpose of the investigation was to identify the functional groups specific to each substance by determining their molecular vibrations.

### 2.5. Swelling Ratio and Open Porosity

In tissue engineering, the swelling capacity is an important parameter for assessing the stability of porous wound-dressings, including the ones based on natural polymers (collagen) and synthetic polymers (carboxymethylcellulose), along with their prospective application in burns treatment. This in vitro evaluation gives valuable information on how the dressing will respond in contact with the body fluids and how this interaction can affect the process of cellular differentiation [32].

Hence, 1 L of simulated body fluid (SBF) solution was first prepared, based on the ionic species concentrations from Kokubo’s methodology [33] (Table 2), corresponding to the ionic concentrations from human blood plasma, and following the steps previously described [34].

Small cylinders of 5 mm diameter, from each sample, were first weighed (determining the initial mass *Wi*), then completely soaked in 5 mL of SBF and maintained at 37 °C for various time periods. After each absorption time *t*, the samples were taken off, gently removed the excess fluid from their surface, then weighed again (determining therefore the mass at time *t*). Using the gravimetric method, the swelling ratio was calculated with Formula (1):

(1) $$Swelling\;ratio\;\%=\frac{Wt-Wi}{Wi}a\times 100\%$$

where *Wt* is the sample weight after immersion at time *t* and *Wi* is the weight of the dry sample (the initial mass) [35].

Open porosity was evaluated in ethanol, using liquid dislocation method [36]. Briefly, for each sample, fragments from the porous composite were placed in a cylinder containing a known volume of ethanol (noted *V*_1_). The increased volume resulting after the immersion of the sample was then measured (noted *V*_2_). The soaked fragments were afterwards removed, and the final volume of ethanol from the cylinder was measured (noted *V*_3_). The open porosity was calculated using Formula (2):

(2) $$\mathrm{Porosity}\;\%=\;\frac{V_{1}\;-\;V_{3}}{V_{2}\;-\;V_{3}}a\times 100\%$$

### 2.6. Enzymatic Degradation in Collagenase Solution

Enzymatic degradation is an analysis used to determine in vitro the stability (or the degree of degradability) of the proposed wound-dressings when placed on the skin injury. The collagen degradation occurs when in contact with collagenase and involves the destruction of peptide bonds from its structure. Collagenase is an enzyme normally present at the lesion site, with an important role in the inflammatory stage of the skin regeneration [37].

Enzymatic degradation of the biocomposite samples was evaluated by completely immerse small cylinders of 5 mm diameter from each sample in 5 mL of collagenase solution 0.0025% and maintain at 37 °C for various time periods. To monitor the mass loss, all samples were weighed before (*Wi*) and after soaking at different time intervals (*Wt*), and Formula (3) was used:

(3) $$\mathrm{Weight}\;\mathrm{loss}\;\%=\frac{Wi-Wt}{Wi}\times 100\%$$

where *Wi* represents the initial mass, and *Wt* represents the mass of the sample at time *t* [38].

### 2.7. Antimicrobial Assay

The strains used for this study, *Escherichia coli ATCC 25922*, *Staphylococcus aureus ATCC 25923*, and *Candida albicans ATCC 10231*, were obtained from the Microbiology laboratory’s collection, Faculty of Biology, University of Bucharest. The selection of these species was carried out for the analysis of the antimicrobial effect of collagen, carboxymethylcellulose, and silver-based wound-dressings by qualitative and quantitative assays.

#### 2.7.1. Inhibition Zone Diameter Assay

The material containing the antimicrobial agent (samples of 0.6 cm in diameter) was sterilized by exposure to UV radiation for 20 min on each side. Then, samples were placed using a sterile clamp on a Petri dish containing Mueller Hinton medium swab inoculated with a microbial suspension of 1–3 × 10^8^ CFU/mL (CFU—Colony Forming Units), corresponding to 0.5 MacFarland density standard. Sabourand Dextrose Agar was used for the growth of yeast strain. The Petri dishes containing microbial culture and evaluated composite samples were incubated for 24 h at 37 °C. Then, the inhibition zone diameter is measured and results are expressed in mm.

#### 2.7.2. Planktonic Growth Rate

Samples from the sterile material were introduced in plates with 24 sterile wells and 1 mL of nutritive broth was added. In the next stage, 20 µL of bacterial suspension with a density of 1–3 × 10^8^ CFU/mL was prepared in physiologic and sterile water, added to the plates and incubated for 24 h. For the analysis of planktonic growth rate, 200 µL of culture from the previous plates were transferred to a plate with 96 wells. The absorbance (at 600 nm) of the obtained cultures was measured with a spectrophotometer, and the obtained optical densities represent the growth degree of planktonic cultures in the presence of the material.

### 2.8. Cell Toxicity Studies

#### 2.8.1. Cell Viability Assessment

The toxicity of Coll CMC Ag biocomposites was evaluated by the CellTiter assay (Promega, Madison, WI, USA). The HaCaT cells were maintained in Dulbecco’s Modified Eagle Medium (DMEM): F12 (Thermo Fisher Scientific Inc., Waltham, MA, USA) supplemented with 10% heat inactivated fetal bovine serum (FBS). About 1 × 10^4^ HaCaT cells were seeded in a 96-well plate (TPP, Trasadingen, Switzerland) with DMEM: F12 containing 10% FBS and maintained at 37 °C in a humidified incubator Heracell 150i CO_2_ incubator (Thermo Fisher Scientific Inc., Waltham, MA, USA) supplemented with 5% CO_2_. The impregnated porous materials were cut with a biopsy driller with a diameter of 4, 6, and 8 mm, and added over the HaCaT cells. The sponge and media were removed after 24 h/72 h of treatment and fresh media containing CellTiter was added. The formazan production was quantified using a multi-well plate reader (TriStar² LB 942 Multimode Microplate Reader, Berthold, Germany) at 570 nm absorbance.

#### 2.8.2. Propidium Iodide (PI) Staining and Cell Cycle Analysis

The cells maintained or not together with Coll CMC Ag were collected in a 5 mL tube and fixed at −20 °C with 70% cold ethanol for at least 24 h. Cells were washed in phosphate saline buffer and incubated with 50 μg/mL PI, 0.2 mg/mL RNase A in PBS for 2 h at 37 °C in the dark. After incubation, PI fluorescence was analyzed on a Beckman Coulter flow cytometer (Beckman Coulter, Brea, CA, USA), and the percentage of cells in each stage of the cell cycle were analyzed using FlowJo 7.2.5 software.

### 2.9. Statistical Analysis

All the experiments were done in triplicates. Data is represented as mean ± standard deviation (S.D.). The graphs and statistical analysis were performed using MS Excel software. Data was compared using one-way analysis of variance (ANOVA), followed by a two tails t-test with Bonferroni post-hoc correction. Values of *p* < (0.05/*n*) were considered statistically significant.

## 3. Results and Discussions

**Figure 1:**
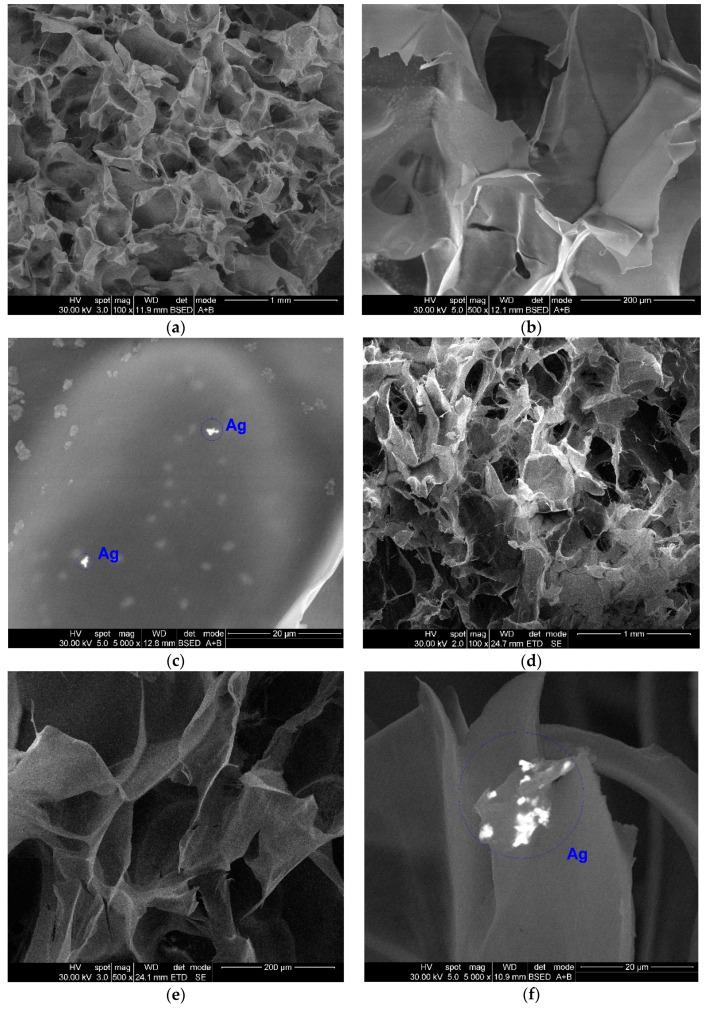

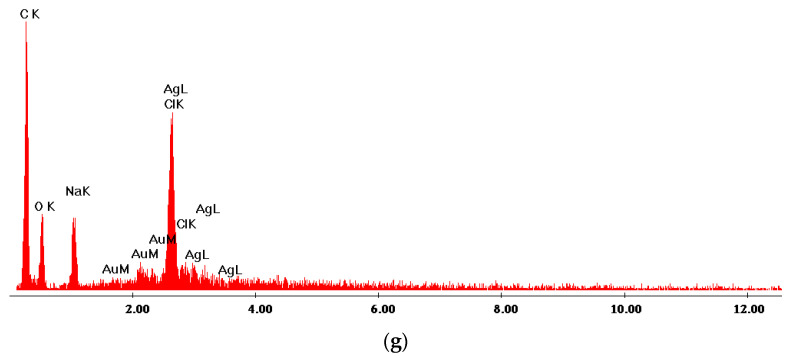
SEM (Scanning Electron Microscopy) micrographs showing the porous morphology and silver distribution from Coll CMC Ag 10 N (**a**–**c**) and Coll CMC Ag 10 G (**d**–**f**) samples, along with the elemental composition (energy dispersive spectrometer (EDS) spectrum) of Coll CMC Ag 10 N (**g**) wound dressing.

SEM images associated to the biocomposite wound dressings with smaller colloidal silver content are presented in Figure 1 (Coll CMC Ag 10 N (a–c), Coll CMC Ag 10 G (d–f)).

The results indicate a three-dimensional porous structure of the scaffolds, characteristic to the freeze-drying method, slightly higher for the non-cross-linked samples. The pores are interconnected, with irregular morphologies and dimensions varying from 100 to 300 μm, better observed from the general images at low magnification (a,d). Moreover, using backscattered electrons detector, nanoparticle agglomerates can be observed in both samples, at the surface of the polymer fibers and sheets (c,f) conferring antimicrobial activity of these samples. The EDS spectrum presented in Figure 1g confirms the silver nature of these particles, along with the others elements characteristic to the sample composition or sample preparation technique.

**Figure 2:**
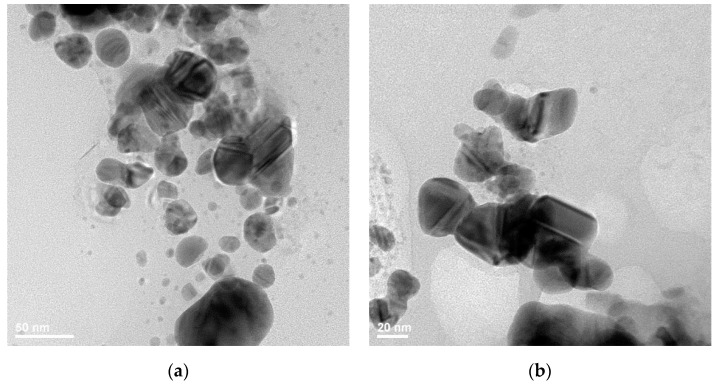

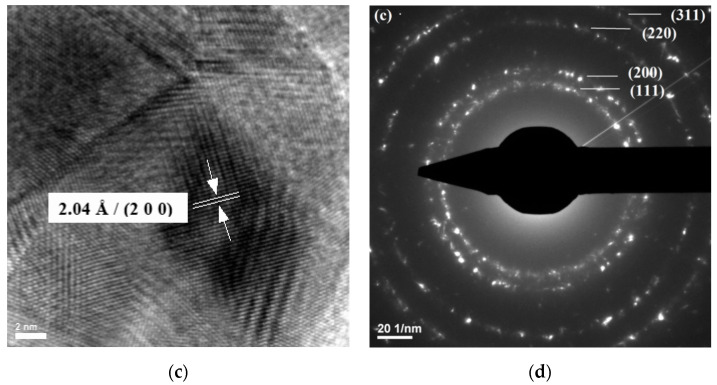
Transmission Electron Microscopy (TEM) images (**a**,**b**); High-Resolution Transmission Electron Microscopy (HR-TEM) image (**c**); and Selected Area Electron Diffraction (SAED) pattern (**d**) for the 100 ppm colloidal silver.

Furthermore, the morphological and structural properties of the identified silver nanoparticles were investigated by transmission electron microscopy, and the resulted TEM, HR-TEM, and SAED images are presented in Figure 2a–d. Thus, AgNPs have truncated triangular and polyhedral morphologies, with a high tendency to form agglomerates due to their reduced dimensions. The obtained particles are highly crystalline, with ordered plans of atoms and the interplanar distance of 2.04 Å (200) (Figure 2c). SAED image (Figure 2d) presents the characteristic silver diffraction rings, with the associated Miller indices of (111), (200), (220), (311), suggesting once more the crystalline nature of the synthesized silver colloids.

Literature studies dedicated to the strong relation between AgNP synthesis conditions, size, morphology, and antimicrobial activity suggest that truncated triangular silver nanoplates with a (111) lattice plane as the basal plane display the strongest biocidal action, compared with spherical and rod-shaped nanoparticles and with Ag^+^ [39,40,41]. Additionally, the same truncated triangular morphology was demonstrated to have the slowest skin penetration capability, making it the ideal solution for antimicrobial skin wound-dressings [42].

**Figure 3:**
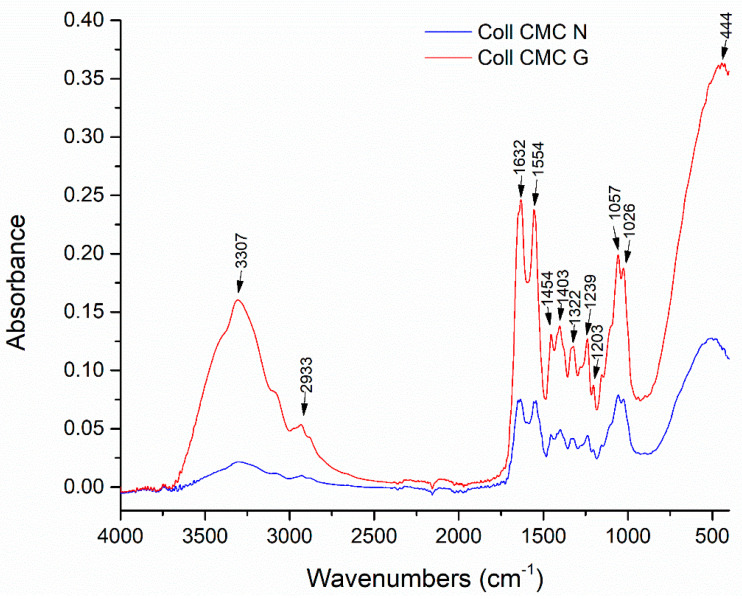
FT-IR spectra for the Coll CMC N and Coll CMC G porous composites.

The Fourier-transform infrared spectroscopy (FT-IR) spectra of the silver free, cross-linked with glutaraldehyde (G) and non-cross-linked (N) collagen-carboxymethylcellulose porous composites are shown in Figure 3. In both spectra are identified similar bands, characteristic to Coll and CMC, with different absorbance intensities, depending on the chemical reticulation process. The presence of the amide I band (C=O stretching) is observed at 1632 cm^−1^. The absorption bands characteristic to amide II (N-H bending) and III are observed at 1554 cm^−1^ and at 1239 cm^−1^, respectively, corresponding to the vibration of the N-H group coupled with the stretching vibrations of C-N and C-H. Additionally, the stretching of −OH and –CH_3_ groups characteristic to CMC were observed at 3307 cm^−1^ and 2933 cm^−1^, while the bands at 1322 and 1403 cm^−1^ represent C=O and C–O stretching vibration of the ionized carboxyl group. In contrast, the Coll CMC N shows these bands at slightly lower wavenumbers and with an accentuated decrease in absorbance, indicating that crosslinking was successful and significantly changed the molecular structure of the polymers, due to the glutaraldehyde −CHO groups reaction with the amino group of collagen [43,44].

**Figure 4:**
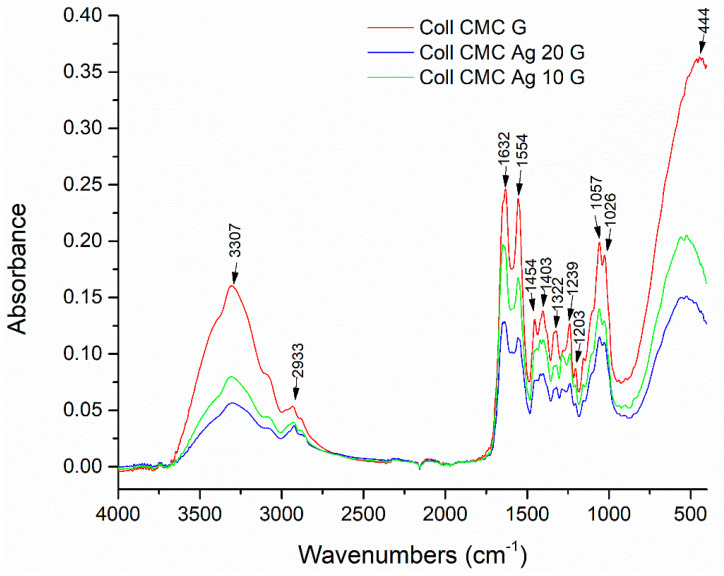
FT-IR (Fourier-transform Infrared Spectroscopy) spectra for the Coll CMC G, Coll CMC Ag 10 G, and Coll CMC Ag 20 G porous composites.

In Figure 4 are comparatively presented the FT-IR spectra of the previously described silver free (Coll CMC G) sample and the silver loaded (Coll CMC Ag 10 G, Coll CMC Ag 20 G), glutaraldehyde cross-linked biocomposites. In the case of Ag-containing samples, changes in position and absorbance of the collagen-specific bands are presented, accentuated by the increase in silver concentration. This can be associated to the collagen interaction with AgNPs, since a potential stabilization of silver nanoparticles with the COO^−^ and NH_2_^+^ groups in collagen is already described in literature [45]. Moreover, cellulose is also known to bind electropositive transition metal atoms by electrostatic interactions [46].

**Figure 5:**
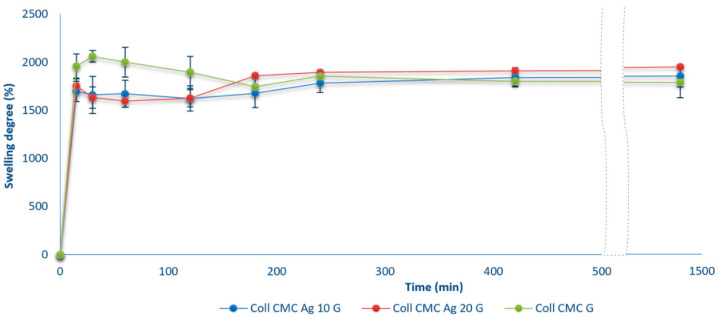
The swelling capacity of cross-linked wound dressing materials during 24 h (presented as mean ± S.D. of 3 replicates).

**Figure 6:**
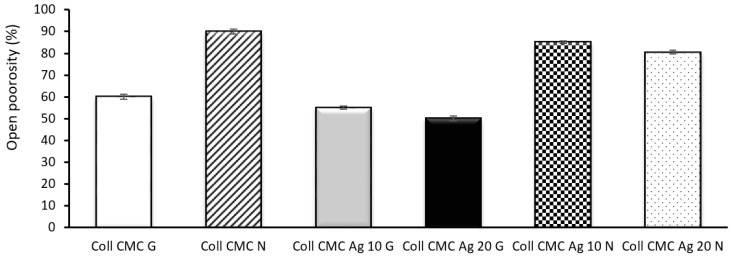
Open porosity (%) evaluation of wound dressing materials (presented as mean ± S.D. of 3 replicates).

The swelling degree (as can be seen in Figure 5) is closely related to the open porosity evaluation results (presented in Figure 6) and mainly expresses the influence of the cross-linking process and silver addition to the main polymeric matrix. Even though the non-cross-linked samples (Coll CMC N, Coll CMC Ag 10 N, and Coll CMC Ag 20 N) have the highest porosity, they were not stable enough to evaluate their swelling capacity through 24 h, losing their integrity after only 2 h, and they were excluded from the final results. Thus, the best swelling capacity after 24 h is assigned to the Coll CMC Ag 20 G sample, which presented a swelling degree of 1900%. For all the samples, an initial fast swelling is observed, practically the highest amount of SBF being absorbed in the first 1–2 h, but afterwards their mass quickly stabilized and maintained until the end of the experiment. This fast swelling is induced by the high porosity, important because these wound dressing can also assure a fast adsorption of the exudates, and thus a proper humidity of the wound can be assured in tens of minutes. The equilibrium differences between the Coll CMC G, Coll CMC Ag 10 G, and Coll CMC Ag 20 G are not statistically significant, therefore we can assume that AgNPs addition has no impact to the porosity and swelling capacity of the biocomposites.

The open porosity of all cross-linked biocomposites was in range of ~50–60%, while the non-cross-linked materials registered open porosity values were in range of ~80–90% (Figure 6). Porosity of a scaffold is crucial for an efficient wound healing, especially because it allows the oxygen permeation and nutriments delivery to the affected area, the best value presented in the literature being in the range of 60–90% [47]

**Figure 7:**
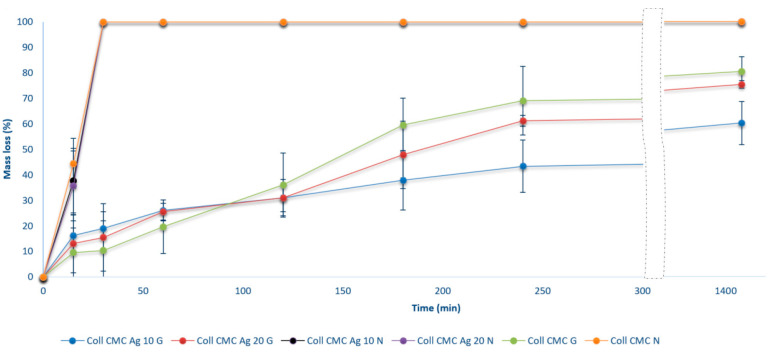
In vitro enzymatic degradation of wound dressing materials during 24 h (presented as mean ± S.D. of 3 replicates).

Subsequently, the samples degradation in a solution of collagenase, a collagen-degrading enzyme, was evaluated. The mass loss for the biocomposite samples is presented in Figure 7. An increase in mass loss was observed over the 24 h for all the cross-linked samples, along with a rapid and total disintegration of the non-cross-linked ones in the first hour of immersion in collagenase. The mass loss rate is slightly increased in the first hour for the samples containing silver nanoparticles, which can be associated with an enhanced collagen structure disintegration. Nevertheless, after 24 h, the mass loss varied between ~60–100%, and it was smaller for the cross-linked, less porous samples, in accordance with previous swelling and porosity results.

As for the obtained results, the obtained dressings resist enzymatic degradation for about 24 h, until they disintegrate. Since it is recognized that microorganisms may produce degrading enzymes for such collagen-based materials [48,49], the antimicrobial experiments aiming to evaluate their efficiency were performed only at 24 h incubation. Moreover, in wound care, dressings are replaced after a few hours—up to 24 h, to reduce the risk of microbial contamination in the wound area [50].

**Figure 8:**
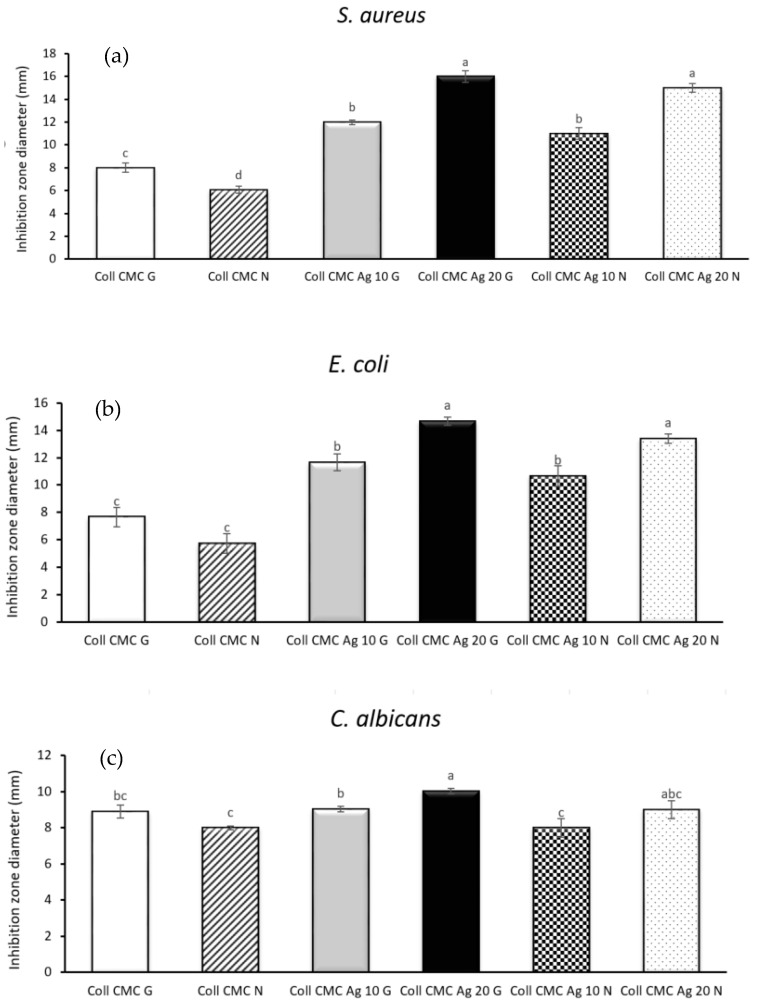
Inhibition zone diameter evaluation for all biocomposite samples against (**a**) *S. aureus*, (**b**) *E. coli*, (**c**) *C. albicans* (presented as mean ± S.D. of 3 replicates); different letters indicate significant differences between each sample. *p* < 0.05/*n* (*n* = 15).

Figure 8 highlights the inhibition zone diameters registered after 24 h of incubation of all biocomposite samples in Gram-negative *E. coli* and Gram-positive *S. aureus* bacteria cultures, as well as in the presence of *C. albicans* yeast strain. Thus, in the case of *S. aureus* and *E. coli* the most effective dressing is Coll CMC Ag 20 G, which exhibits inhibition zone diameters around 16 and 15 mm, respectively. The control samples (Coll CMC G and Coll CMC N) generated the smallest inhibitory zone, due to the lack of antimicrobial agent. The obtained composite materials exhibit a better antibacterial effect (against both *S. aureus* and *E. coli*) than antifungal effect (against *C. albicans*). There were no statistically significant differences between the samples containing similar amounts of silver nanoparticles (e.g., Coll CMC Ag 20 G/Coll CMC Ag 20 N or Coll CMC Ag 10 G/Coll CMC Ag 10 N), no matter the microbial strain. These results also confirm that the antimicbrobial potential of the evaluated composite materials is proportional with the amount of AgNPs contained in the material [51].

**Figure 9:**
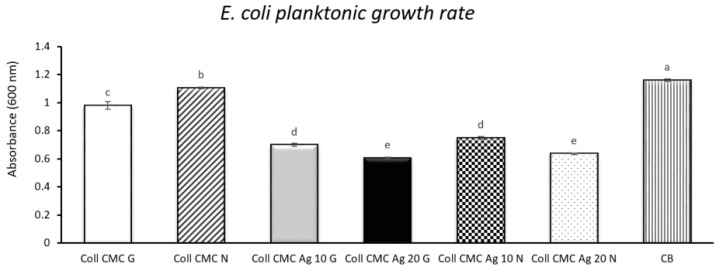
*E. coli* planktonic growth rate evaluation for all biocomposite samples (presented as mean ± S.D. of 3 replicates); CB = bacterial control containing no composite material; different letters indicate significant differences between each sample. *p* < 0.05/*n* (*n* = 21).

Figure 9 presents the planktonic growth rate of *E. coli* (expressed as the absorbance values measured at 600 nm) when in contact with the biocomposite wound-dressings, along with the absorbance registered for the bacterial control (containing *E. coli* untreated culture). Thus, the lowest absorbance value, correlated with the best antibacterial activity is attributed to the sample Coll CMC Ag 20 G. The silver free samples (Coll CMC G and Coll CMC N) have a reduced antibacterial activity, correlated with absorbance values similar to the value of the bacterial untreated control. The quantitative analysis results are in accordance with the ones from the qualitative assay and with the literature studies, which suggest an enhanced antimicrobial activity directly proportional with the AgNPs content from the samples [52]. Similar to the qualitative antimicrobial test, no statistically significant differences between the samples containing similar amounts of silver nanoparticles were found.

The obtained results are in accordance with other studies reporting the great antimicrobial efficiency of AgNPs, when they are integrated in various polymeric matrices [53,54] and coatings, including wound-dressings [24,55]. The antibactericidal properties of silver are well known; briefly, their mechanism relays on the generation of reactive ions which are able to interfere with various cellular biomolecules, such as lipids, proteins, and even nucleic acids. AgNPs kill bacteria through numerous means: (i) Perforate or disrupt cell membranes, (ii) induce the generation of reactive oxygen species (ROS), which are responsible for cell lysis and interference with vital biomolecules, (iii) may interfere with ribosome function, altering translation, and (iv) inhibit DNA replication [56]. The antimicrobial effect of AgNPs may be different in microscopic fungi, especially due to the cellular wall and nature of these microorganisms. However, such NPs could be tailored to efficiently target yeasts and pathogenic fungi [57,58].

**Figure 10:**
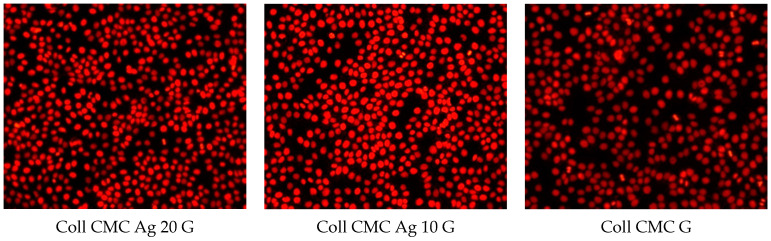

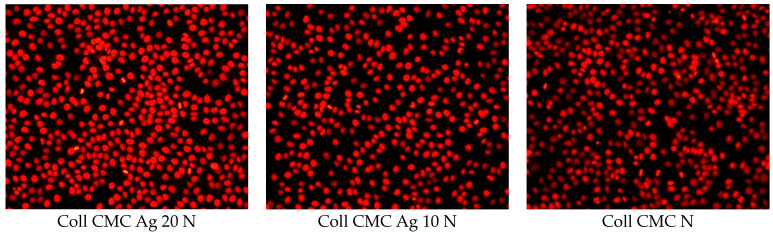
Micrographs showing the morphology of HaCaT cells grown in the presence of Coll CMC with various AgNPs concentration or silver free (control) nanomodified biocomposites. The porous materials were cut with a biopsy driller with a diameter of 4 mm before impregnation.

**Figure 11:**
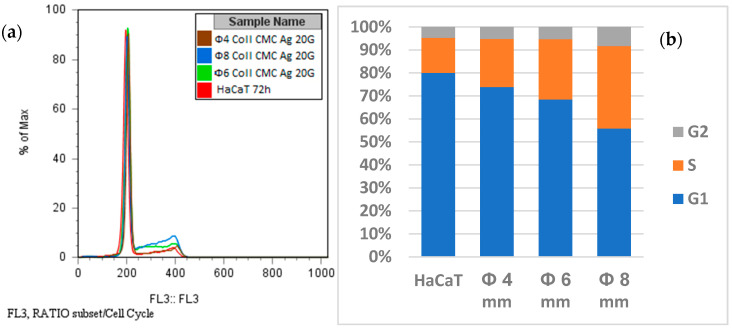
The cell cycle analysis of HaCaT cells maintained for 72 h with Coll CMC Ag 20G impregnated sample: (**a**) Overlayed histogram, (**b**) graphical analysis; the porous materials were cut with a biopsy driller with a diameter of 4, 6, and 8 mm before impregnation.

After analysing antimicrobial results, we performed the in vitro cytotoxicity assessment (Figure 10 and Figure 11).

The 24-h cell viability assessment using the CellTiter kit showed that HaCaT cells were not affected by the presence of the analyzed Coll CMC Ag samples. This observation was also supported by the propidium iodide stain. Cell morphology and their adherent state are maintained, the fluorescent microscopy analysis showing that their aspect is similar to the untreated control (Figure 10). On the other hand, the cell cycle evaluation showed a slight increase in the S and G2/M phases at 72 h (Figure 11). These increases were correlated with the increase of the diameter of the tested biocomposite and with the increase of the silver in the product, respectively. It was already noticed that the silver nanoparticles induce G2/M cell cycle arrest and strong toxicity [59,60]. However, the accumulation of cells in the subG1 phase associated with the irreversible damage and cell death was not observed in our analysis. That means that the obtained biocomposites can be used in long time treatments, with no cytotoxic effects.

Our data reveal that the designed composite materials could be efficiently utilized as antimicrobial dressings, with stable biological properties and no cytotoxicity for at least 24 h.

## 4. Conclusions

The combination of AgNPs with the traditional antimicrobial treatment strategy represents great opportunities in nanomedicine, especially in the case of burns and chronic wounds because they can considerably affect the lives of patients, sometimes leading to death. By adding silver nanoparticles to a biocomposite material based on natural and synthetic polymers, innovative dressings are obtained and provide an ideal environment to avoid further development of microbial infections. Thus, freeze-dried collagen-carboxymethylcellulose biocomposite dressings with various amounts of 100 ppm colloidal silver nanoparticle solution were obtained. Ag was chemically synthesized through an effective and easy method, resulting nanoparticles with truncated triangular and polyhedral morphologies. Based on the information provided by the swelling ratio, open porosity and enzymatic degradation capacity, it has been confirmed that the cross-linked dressings absorbed a greater amount of SBF and they were more difficult to degrade in a collagenase solution. The porous nature of the obtained biocomposites was confirmed through SEM analysis, highlighting the interconnected collagen fibers and micrometric pores, with homogenous distribution of silver nanoparticles in the polymeric matrix. Both qualitative and quantitative assessments of the antimicrobial potential revealed that the silver containing materials inhibit the growth of bacteria more than fungi, with enhanced activity against *E. coli* and *S. aureus*.

Our results support the idea that the nanostructured composite polymeric materials containing AgNPs are biocompatible and thus successful candidates for the development of efficient dressings, which may assist the therapy of various wounds. Further studies will be focused on determining the release kinetics of silver ions and the associated antimicrobial potential of the obtained solutions.

## Figures and Tables

**Table 1:** The composition of biocomposite wound dressings. AgNPs: Silver nanoparticles; CMC: Carboxymethylcellulose.

Sample Name	100 ppm Colloidal AgNPs Solution (mL)	Calculated AgNPs Concentration in the Dried Sample (w/w %)	Glutaraldehyde 0.025% (mL)
Coll CMC Ag 20 G	20	0.02	5
Coll CMC Ag 10 G	10	0.01	5
Coll CMC G	0	0	5
Coll CMC Ag 20 N	20	0.02	0
Coll CMC Ag 10 N	10	0.01	0
Coll CMC N	0	0	0

**Table 2:** Ionic species concentrations used in simulated body fluid (SBF) preparation [33].

Ionic Species Concentrations (mmol/L)										
**SBF Solution**	**Na^+^**	**K^+^**	**Mg^2+^**	**Ca^2+^**	**Cl^−^**	**HCO_3_^−^**	**HPO_4_^2−^**	**SO_4_^2−^**	**Buffer Solution**	pH
	142.0	5.0	1.5	2.5	147.8	4.2	1.0	0.5		7.25

## Data Availability

Not applicable.

## References

[1] Benítez J.M., Montáns F.J. (2017). The mechanical behavior of skin: Structures and models for the finite element analysis. Comput. Struct..

[2] Lai-Cheong J.E., McGrath J.A. (2017). Structure and function of skin, hair and nails. Medicine.

[3] Kaminsky D. (2011). The Netter Collection of Medical Illustrations: Respiratory System.

[4] Negut I., Grumezescu V., Grumezescu A.M. (2018). Treatment Strategies for Infected Wounds. Molecules.

[5] Bayramov D.F., Neff J.A. (2017). Beyond conventional antibiotics—New directions for combination products to combat biofilm. Adv. Drug Deliv. Rev..

[6] Ghosh C., Sarkar P., Issa R., Haldar J. (2019). Alternatives to Conventional Antibiotics in the Era of Antimicrobial Resistance. Trends Microbiol..

[7] Smith R., Russo J., Fiegel J., Brogden N. (2020). Antibiotic Delivery Strategies to Treat Skin Infections When Innate Antimicrobial Defense Fails. Antibiotics.

[8] Amanzadi B., Mirzaei E., Hassanzadeh G., Mahdaviani P., Boroumand S., Abdollahi M., Hosseinabdolghaffari A., Majidi R.F. (2019). Chitosan-based layered nanofibers loaded with herbal extract as wound-dressing materials on wound model studies. Biointerface Res. Appl. Chem..

[9] Saghazadeh S., Rinoldi C., Schot M., Kashaf S.S., Sharifi F., Jalilian E., Nuutila K., Giatsidis G., Mostafalu P., Derakhshandeh H. (2018). Drug delivery systems and materials for wound healing applications. Adv. Drug Deliv. Rev..

[10] Abdelghany A.M., Meikhail M.S., El-Bana A.A. (2019). Microbial Activity and Swelling Behavior of Chitosan/Polyvinyl Alcohol/Sodium Alginate Semi-Natural Terpolymer Interface Containing Amoxicillin for Wound Dressing Applications. Biointerface Res. Appl. Chem..

[11] Gokarneshan N. (2019). Application of natural polymers and herbal extracts in wound management. Advanced Textiles for Wound Care.

[12] Nuutila K., Eriksson E. (2020). Moist Wound Healing with Commonly Available Dressings. Advances in Wound Care.

[13] Rangel U.J.S., Oda H., Akerman J., Wang Z., Chang J., Fox P.M. (2020). Topical Antibiotic Elution in a Collagen Rich Hydrogel for Healing of Infected Wounds. Plast. Reconstr. Surg. Glob. Open.

[14] Kanikireddy V., Varaprasad K., Jayaramudu T., Karthikeyan C., Sadiku R. (2020). Carboxymethyl cellulose-based materials for infection control and wound healing: A review. Int. J. Biol. Macromol..

[15] Koneru A., Dharmalingam K., Anandalakshmi R. (2020). Cellulose based nanocomposite hydrogel films consisting of sodium carboxymethylcellulose–grapefruit seed extract nanoparticles for potential wound healing applications. Int. J. Biol. Macromol..

[16] Simões D., Miguel S.P., Ribeiro M.P., Coutinho P., Mendonça A.G., Correia I.J. (2018). Recent advances on antimicrobial wound dressing: A review. Eur. J. Pharm. Biopharm..

[17] Koehler J., Brandl F.P., Goepferich A.M. (2018). Hydrogel wound dressings for bioactive treatment of acute and chronic wounds. Eur. Polym. J..

[18] Andersson D.I., Balaban N.Q., Baquero F., Courvalin P., Glaser P., Gophna U., Kishony R., Molin S., Tønjum T. (2020). Antibiotic resistance: Turning evolutionary principles into clinical reality. FEMS Microbiol. Rev..

[19] Ng V.W., Chan J.M., Sardon H., Ono R.J., García J.M., Yang Y.Y., Hedrick J.L. (2014). Antimicrobial hydrogels: A new weapon in the arsenal against multidrug-resistant infections. Adv. Drug Deliv. Rev..

[20] Neacsu I.-A., Melente A.E., Holban A.-M., Ficai A., Ditu L.-M., Kamerzan C.-M., Tihăuan B.M., Nicoara A.I., Bezirtzoglou E., Chifiriuc M.-C. (2019). Novel hydrogels based on collagen and ZnO nanoparticles with antibacterial activity for improved wound dressings. Rom. Biotechnol. Lett..

[21] Paduraru A., Ghitulica C., Trusca R., Surdu V.A., Neacsu I.A., Holban A.M., Birca A.C., Iordache F., Vasile B.S. (2019). Antimicrobial Wound Dressings as Potential Materials for Skin Tissue Regeneration. Materials.

[22] Wahid F., Zhong C., Wang H.-S., Hu X.-H., Chu L.-Q. (2017). Recent Advances in Antimicrobial Hydrogels Containing Metal Ions and Metals/Metal Oxide Nanoparticles. Polymers.

[23] Deshmukh S., Patil S., Mullani S., Delekar S. (2019). Silver nanoparticles as an effective disinfectant: A review. Mater. Sci. Eng. C.

[24] Ali G.W., Abd El-Moez S.H., Abdel-Fattah W.A. (2019). Synthesis and characterization of nontoxic silver nano-particles with preferential bactericidal activity. Biointerface Res. Appl. Chem..

[25] Stoica A.E., Chircov C., Grumezescu A.M. (2020). Hydrogel Dressings for the Treatment of Burn Wounds: An Up-To-Date Overview. Materials.

[26] Zielińska A., Skwarek E., Zaleska A., Gazda M., Hupka J. (2009). Preparation of silver nanoparticles with controlled particle size. Procedia Chem..

[27] Demchenko V., Riabov S., Kobylinskyi S., Goncharenko L., Rybalchenko N., Kruk A., Moskalenko O., Shut M. (2020). Effect of the type of reducing agents of silver ions in interpolyelectrolyte-metal complexes on the structure, morphology and properties of silver-containing nanocomposites. Sci. Rep..

[28] Djokić S. (2008). Synthesis and Antimicrobial Activity of Silver Citrate Complexes. Bioinorg. Chem. Appl..

[29] Wang H., Qiao X., Chen J., Wang X., Ding S. (2005). Mechanisms of PVP in the preparation of silver nanoparticles. Mater. Chem. Phys..

[30] Jafari N., Karimi L., Mirjalili M., Derakhshan S.J. (2016). Effect of Silver Particle size on color and Antibacterial properties of silk and cotton Fabrics. Fibers Polym..

[31] Albu M.G. (2011). Collagen Gels and Matrices for Biomedical Applications.

[32] Tsakovska I., Pajeva I., Al Sharif M., Alov P., Fioravanzo E., Kovarich S., Worth A.P., Richarz A.-N., Yang C., Mostrag-Szlichtyng A. (2017). Quantitative structure-skin permeability relationships. Toxicology.

[33] Kokubo T., Kushitani H., Sakka S., Kitsugi T., Yamamuro T. (1990). Solutions able to reproduce in vivo surface-structure changes in bioactive glass-ceramic A-W3. J. Biomed. Mater. Res..

[34] Neacsu I.A., Serban A.P., Nicoara A.I., Trusca R., Ene V.L., Iordache F. (2020). Biomimetic Composite Scaffold Based on Naturally Derived Biomaterials. Polymers.

[35] Ghica M.V., Albu M.G., Popa L., Moisescu S. (2013). Response surface methodology and Taguchi approach to assess the combined effect of formulation factors on minocycline delivery from collagen sponges. Die Pharm..

[36] Sharma C., Dinda A.K., Potdar P.D., Chou C.-F., Mishra N.C. (2016). Fabrication and characterization of novel nano-biocomposite scaffold of chitosan–gelatin–alginate–hydroxyapatite for bone tissue engineering. Mater. Sci. Eng. C.

[37] Das A., Datta S., Roche E., Chaffee S., Jose E., Shi L., Grover K., Khanna S., Sen C.K., Roy S. (2018). Novel mechanisms of Collagenase Santyl Ointment (CSO) in wound macrophage polarization and resolution of wound inflammation. Sci. Rep..

[38] Marin Ș., Albu Kaya M.G., Ghica M.V., Dinu-Pîrvu C., Popa L., Udeanu D.I., Mihai G., Enachescu M. (2018). Collagen-Polyvinyl Alcohol-Indomethacin Biohybrid Matrices as Wound Dressings. Pharmaceutics.

[39] Sadeghi B., Garmaroudi F.S., Hashemi M., Nezhad H., Nasrollahi A., Ardalan S., Ardalan S. (2012). Comparison of the anti-bacterial activity on the nanosilver shapes: Nanoparticles, nanorods and nanoplates. Adv. Powder Technol..

[40] Vasile O.R., Andronescu E., Truşcă R., Vasile E., Holban A.M., Chifiriuc M.C., Iordache F., Maniu H., Bleotu C., Neacşu I.A. (2019). Structure-grain size-synthesis route of silver nanoparticles: A correlation with the cytotoxic effect. Rom. J. Morphol. Embryol..

[41] Khodashenas B., Ghorbani H.R. (2019). Synthesis of silver nanoparticles with different shapes. Arab. J. Chem..

[42] Tak Y.K., Pal S., Naoghare P.K., Rangasamy S., Song J.M. (2015). Shape-Dependent Skin Penetration of Silver Nanoparticles: Does It Really Matter?. Sci. Rep..

[43] Amri M., Firdaus M., Fauzi M., Chowdhury S., Fadilah N., Hamirul W.W., Reusmaazran M., Aminuddin B., Ruszymah B. (2014). Cytotoxic evaluation of biomechanically improved crosslinked ovine collagen on human dermal fibroblasts. Bio-Med. Mater. Eng..

[44] Akhshabi S., Biazar E., Singh V., Keshel S.H., Nagaraja G. (2018). The effect of glutaraldehyde cross-linker on structural and biocompatibility properties of collagen-chondroitin sulfate electrospun mat. Mater. Technol..

[45] Drobotă M., Grierosu I., Radu I., Vasilescu D.S. (2015). The Effect of Silver Nanoparticles on the Collagen Secondary Structure. Key Eng. Mater..

[46] Carbone M., Donia D.T., Sabbatella G., Antiochia R. (2016). Silver nanoparticles in polymeric matrices for fresh food packaging. J. King Saud Univ.-Sci..

[47] Negut I., Dorcioman G., Grumezescu V. (2020). Scaffolds for Wound Healing Applications. Polymers.

[48] Singh B., Fleury C., Jalalvand F., Riesbeck K. (2012). Human pathogens utilize host extracellular matrix proteins laminin and collagen for adhesion and invasion of the host. FEMS Microbiol. Rev..

[49] Harrington D.J. (1996). Bacterial collagenases and collagen-degrading enzymes and their potential role in human disease. Infect. Immun..

[50] Sood A., Granick M.S., Tomaselli N.L. (2014). Wound Dressings and Comparative Effectiveness Data. Adv. Wound Care.

[51] Keshvadi M., Karimi F., Valizadeh S., Valizadeh A. (2019). Comparative study of antibacterial inhibitory effect of silver nanoparticles and garlic oil nanoemulsion with their combination. Biointerface Res. Appl. Chem..

[52] Garibo D., Borbón-Nuñez H.A., De León J.N.D., Mendoza E.G., Estrada I., Toledano-Magaña Y., Tiznado H., Ovalle-Marroquin M., Soto-Ramos A.G., Blanco A. (2020). Green synthesis of silver nanoparticles using Lysiloma acapulcensis exhibit high-antimicrobial activity. Sci. Rep..

[53] Fadlilah D.R., Endarko E., Ratnasari A., Hozairi H., Yusop Z., Syafiuddin A. (2019). Enhancement of antibacterial properties of various polymers functionalized with silver nanoparticles. Biointerface Res. Appl. Chem..

[54] Mandal P., Ghosh S. (2020). Green Approach to the Synthesis of Poly(Vinyl Alcohol)-Silver Nanoparticles Hybrid Using Rice Husk Extract and Study of its Antibacterial Activity. Biointerface Res. Appl. Chem..

[55] Gunell M., Haapanen J., Brobbey K.J., Saarinen J.J., Toivakka M., Mäkelä J.M., Huovinen P., Eerola E. (2017). Antimicrobial characterization of silver nanoparticle-coated surfaces by “touch test” method. Nanotechnol. Sci. Appl..

[56] Yin I.X., Zhang J., Zhao I.S., Mei M.L., Li Q., Chu C.H. (2020). The Antibacterial Mechanism of Silver Nanoparticles and Its Application in Dentistry. Int. J. Nanomed..

[57] Elgorban A.M., El-Samawaty A.E.-R.M., Yassin M.A., Sayed S.R., Adil S.F., Elhindi K.M., Bakri M., Khan M. (2016). Antifungal silver nanoparticles: Synthesis, characterization and biological evaluation. Biotechnol. Biotechnol. Equip..

[58] Mussin J.E., Roldán M.V., Rojas F., Sosa M.D.L. (2019). Ángeles; Pellegri, N.; Giusiano, G. Antifungal activity of silver nanoparticles in combination with ketoconazole against Malassezia furfur. AMB Express.

[59] Lee Y.S., Kim D.W., Oh J.H., Yoon S., Choi M.S., Lee S.K., Kim J.W., Lee K., Song C.-W. (2011). Silver nanoparticles induce apoptosis and G2/M arrest via PKCζ-dependent signaling in A549 lung cells. Arch. Toxicol..

[60] Asharani P.V., Mun G.L.K., Hande M.P., Valiyaveettil S. (2009). Cytotoxicity and Genotoxicity of Silver Nanoparticles in Human Cells. ACS Nano.

